# The metabolic advantage of tumor cells

**DOI:** 10.1186/1476-4598-10-70

**Published:** 2011-06-07

**Authors:** Maurice Israël, Laurent Schwartz

**Affiliations:** 1 Av Aristide Briand 2, Bures sur Yvette 91440, France; 2 LIX: Ecole Polytechnique Palaiseau 91128 and Hôpital Raymond Poincaré, 104 Bd Raymond Poincaré Garches 92380m, France

## Abstract

1- Oncogenes express proteins of "Tyrosine kinase receptor pathways", a receptor family including insulin or IGF-Growth Hormone receptors. Other oncogenes alter the PP2A phosphatase brake over these kinases.

2- Experiments on pancreatectomized animals; treated with pure insulin or total pancreatic extracts, showed that choline in the extract, preserved them from hepatomas.

Since choline is a methyle donor, and since methylation regulates PP2A, the choline protection may result from PP2A methylation, which then attenuates kinases.

3- Moreover, kinases activated by the boosted signaling pathway inactivate pyruvate kinase and pyruvate dehydrogenase. In addition, demethylated PP2A would no longer dephosphorylate these enzymes. A "bottleneck" between glycolysis and the oxidative-citrate cycle interrupts the glycolytic pyruvate supply now provided via proteolysis and alanine transamination. This pyruvate forms lactate (Warburg effect) and NAD+ for glycolysis. Lipolysis and fatty acids provide acetyl CoA; the citrate condensation increases, unusual oxaloacetate sources are available. ATP citrate lyase follows, supporting aberrant transaminations with glutaminolysis and tumor lipogenesis. Truncated urea cycles, increased polyamine synthesis, consume the methyl donor SAM favoring carcinogenesis.

4- The decrease of butyrate, a histone deacetylase inhibitor, elicits epigenic changes (PETEN, P53, IGFBP decrease; hexokinase, fetal-genes-M2, increase)

5- IGFBP stops binding the IGF - IGFR complex, it is perhaps no longer inherited by a single mitotic daughter cell; leading to two daughter cells with a mitotic capability.

6- An excess of IGF induces a decrease of the major histocompatibility complex MHC1, Natural killer lymphocytes should eliminate such cells that start the tumor, unless the fever prostaglandin PGE2 or inflammation, inhibit them...

## Introduction

The metabolic network of biochemical pathways forms a system controlled by a few switches, changing the finality of this system. Specific substrates and hormones control such switches. If for example, glycemia is elevated, the pancreas releases insulin, activating anabolism and oxidative glycolysis, energy being required to form new substance or refill stores. If starvation decreases glycemia, glucagon and epinephrine activate gluconeogenesis and ketogenesis to form nutriments, mobilizing body stores. The different finalities of the system are or oriented by switches sensing the NADH/NAD+, the ATP/AMP, the cAMP/AMP ratios or the O2 supply... We will not describe here these metabolic finalities and their controls found in biochemistry books.

Many of the switches depend of the phosphorylation of key enzymes that are active or not. Evidently, there is some coordination closing or opening the different pathways. Take for example gluconeogenesis, the citrate condensation slows down, sparing OAA, which starts the gluconeogenic pathway. In parallel, one also has to close pyruvate kinase (PK); if not, phosphoenolpyruvate would give back pyruvate, interrupting the pathway. Hence, the properties of key enzymes acting like switches on the pathway specify the finality of the system. Our aim is to show that tumor cells invent a new specific finality, with mixed glycolysis and gluconeogenesis features. This very special metabolism gives to tumor cells a selective advantage over normal cells, helping the tumor to develop at the detriment of the rest of the body.

## I Abnormal metabolism of tumors, a selective advantage

The initial observation of Warburg 1956 on tumor glycolysis with lactate production is still a crucial observation [1]. Two fundamental findings complete the metabolic picture: the discovery of the M2 pyruvate kinase (PK) typical of tumors [2] and the implication of tyrosine kinase signals and subsequent phosphorylations in the M2 PK blockade [3-5].

A typical feature of tumor cells is a glycolysis associated to an inhibition of apoptosis. Tumors over-express the high affinity hexokinase 2, which strongly interacts with the mitochondrial ANT-VDAC-PTP complex. In this position, close to the ATP/ADP exchanger (ANT), the hexokinase receives efficiently its ATP substrate [6,7]. As long as hexokinase occupies this mitochondria site, glycolysis is efficient. However, this has another consequence, hexokinase pushes away from the mitochondria site the permeability transition pore (PTP), which inhibits the release of cytochrome C, the apoptotic trigger [8]. The site also contains a voltage dependent anion channel (VDAC) and other proteins. The repulsion of PTP by hexokinase would reduce the pore size and the release of cytochrome C. Thus, the apoptosome-caspase proteolytic structure does not assemble in the cytoplasm. The liver hexokinase or glucokinase, is different it has less interaction with the site, has a lower affinity for glucose; because of this difference, glucose goes preferentially to the brain.

Further, phosphofructokinase gives fructose 1-6 bis phosphate; glycolysis is stimulated if an allosteric analogue, fructose 2-6 bis phosphate increases in response to a decrease of cAMP. The activation of insulin receptors in tumors has multiple effects, among them; a decrease of cAMP, which will stimulate glycolysis.

Another control point is glyceraldehyde P dehydrogenase that requires NAD+ in the glycolytic direction. If the oxygen supply is normal, the mitochondria malate/aspartate (MAL/ASP) shuttle forms the required NAD+ in the cytosol and NADH in the mitochondria. In hypoxic conditions, the NAD+ will essentially come via lactate dehydrogenase converting pyruvate into lactate. This reaction is prominent in tumor cells; it is the first discovery of Warburg on cancer.

At the last step of glycolysis, pyruvate kinase (PK) converts phosphoenolpyruvate (PEP) into pyruvate, which enters in the mitochondria as acetyl CoA, starting the citric acid cycle and oxidative metabolism. To explain the PK situation in tumors we must recall that PK only works in the glycolytic direction, from PEP to pyruvate, which implies that gluconeogenesis uses other enzymes for converting pyruvate into PEP. In starvation, when cells need glucose, one switches from glycolysis to gluconeogenesis and ketogenesis; PK and pyruvate dehydrogenase (PDH) are off, in a phosphorylated form, presumably following a cAMP-glucagon-adrenergic signal. In parallel, pyruvate carboxylase (Pcarb) becomes active. Moreover, in starvation, much alanine comes from muscle protein proteolysis, and is transaminated into pyruvate. Pyruvate carboxylase first converts pyruvate to OAA and then, PEP carboxykinase converts OAA to PEP etc..., until glucose. The inhibition of PK is necessary, if not one would go back to pyruvate. Phosphorylation of PK, and alanine, inhibit the enzyme.

Well, tumors have a PK and a PDH inhibited by phosphorylation and alanine, like for gluconeogenesis, in spite of an increased glycolysis! Moreover, in tumors, one finds a particular PK, the M2 embryonic enzyme [2,9,10] the dimeric, phosphorylated form is inactive, leading to a "bottleneck ". The M2 PK has to be activated by fructose 1-6 bis P its allosteric activator, whereas the M1 adult enzyme is a constitutive active form. The M2 PK bottleneck between glycolysis and the citric acid cycle is a typical feature of tumor cell glycolysis.

We also know that starvation mobilizes lipid stores from adipocyte to form ketone bodies, they are like glucose, nutriments for cells. Growth hormone, cAMP, AMP, activate a lipase, which provides fatty acids; their β oxidation cuts them into acetyl CoA in mitochondria and in peroxisomes for very long fatty acids; forming ketone bodies. Normally, citrate synthase slows down, to spare acetyl CoA for the ketogenic route, and OAA for the gluconeogenic pathway. Like for starvation, tumors mobilize lipid stores. But here, citrate synthase activity is elevated, condensing acetyl CoA and OAA [11-13]; citrate increases, ketone bodies decrease. Consequently, ketone bodies will stop stimulating Pcarb. In tumors, the OAA needed for citrate synthase will presumably come from PEP, via reversible PEP carboxykinase or other sources. The quiescent Pcarb will not process the pyruvate produced by alanine transamination after proteolysis, leaving even more pyruvate to lactate dehydrogenase, increasing the lactate released by the tumor, and the NAD+ required for glycolysis. Above the bottleneck, the massive entry of glucose accumulates PEP, which converts to OAA via mitochondria PEP carboxykinase, an enzyme requiring biotine-CO2-GDP. This source of OAA is abnormal, since Pcarb, another biotin-requiring enzyme, should have provided OAA. Tumors may indeed contain "morule inclusions" of biotin-enzyme [14] suggesting an inhibition of Pcarb, presumably a consequence of the maintained citrate synthase activity, and decrease of ketone bodies that normally stimulate Pcarb. The OAA coming via PEP carboxykinase and OAA coming from aspartate transamination or via malate dehydrogenase condenses with acetyl CoA, feeding the elevated tumoral citric acid condensation starting the Krebs cycle. Thus, tumors have to find large amounts of acetyl CoA for their condensation reaction; it comes essentially from lipolysis and β oxidation of fatty acids, and enters in the mitochondria via the carnitine transporter. This is the major source of acetyl CoA; since PDH that might have provided acetyl CoA remains in tumors, like PK, in the inactive phosphorylated form. The blockade of PDH [15] was recently reversed by inhibiting its kinase [16,17]. The key question is then to find out why NADH, a natural citrate synthase inhibitor did not switch off the enzyme in tumor cells. Probably, the synthesis of NADH by the dehydrogenases of the Krebs cycle and malate/aspartate shuttle, was too low, or the oxidation of NADH via the respiratory electron transport chain and mitochondrial complex1 (NADH dehydrogenase) was abnormally elevated. Another important point concerns PDH and α ketoglutarate dehydrogenase that are homologous enzymes, they might be regulated in a concerted way; when PDH is off, α ketoglutarate dehydrogenase might be also be slowed. Moreover, this could be associated to an upstream inhibition of aconinase by NO, or more probably to a blockade of isocitrate dehydrogenase, which favors in tumor cells, the citrate efflux from mitochondria, and the ATP citrate lyase route. Normally, an increase of NADH inhibits the citrate condensation, favoring the ketogenic route associated to gluconeogenesis, which turns off glycolysis. Apparently, this regulation does not occur in tumors, since citrate synthase remains active. Moreover, in tumor cells, the α ketoglutarate not processed by α ketoglutarate dehydrogenase converts to glutamate, via glutamate dehydrogenase, in this direction the reaction forms NAD+, backing up the LDH production. Other sources of glutamate are glutaminolysis, which increases in tumors [2].

**Figure 1:**
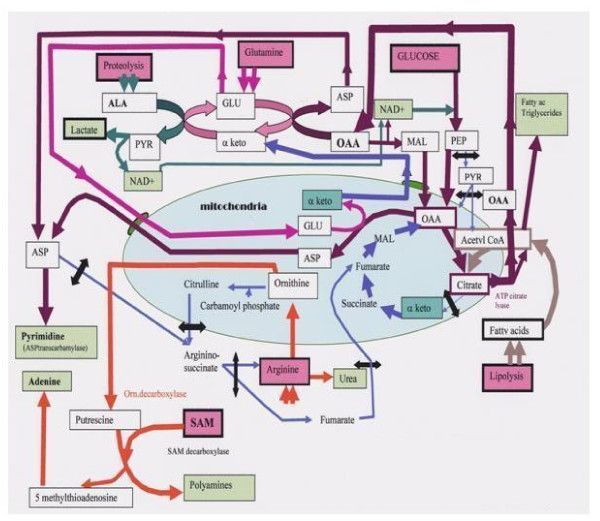
**Cancer metabolism**. Glycolysis is elevated in tumors, but a pyruvate kinase (PK) "bottleneck" interrupts phosphoenol pyruvate (PEP) to pyruvate conversion. Thus, alanine following muscle proteolysis transaminates to pyruvate, feeding lactate dehydrogenase, converting pyruvate to lactate, (Warburg effect) and NAD+ required for glycolysis. Cytosolic malate dehydrogenase also provides NAD+ (in OAA to MAL direction). Malate moves through the shuttle giving back OAA in the mitochondria. Below the PK-bottleneck, pyruvate dehydrogenase (PDH) is phosphorylated (second bottleneck). However, citrate condensation increases: acetyl-CoA, will thus come from fatty acids β-oxydation and lipolysis, while OAA sources are via PEP carboxy kinase, and malate dehydrogenase, (pyruvate carboxylase is inactive). Citrate quits the mitochondria, (note interrupted Krebs cycle). In the cytosol, ATPcitrate lyase cleaves citrate into acetyl CoA and OAA. Acetyl CoA will make fatty acids-triglycerides. Above all, OAA pushes transaminases in a direction usually associated to gluconeogenesis! This consumes protein stores, providing alanine (ALA); like glutamine, it is essential for tumors. The transaminases output is aspartate (ASP) it joins with ASP from the shuttle and feeds ASP transcarbamylase, starting pyrimidine synthesis. ASP in not processed by argininosuccinate synthetase, which is blocked, interrupting the urea cycle. Arginine gives ornithine via arginase, ornithine is decarboxylated into putrescine by ornithine decarboxylase. Putrescine and SAM form polyamines (spermine spermidine) via SAM decarboxylase. The other product 5-methylthioadenosine provides adenine. Arginine deprivation should affect tumors. The SAM destruction impairs methylations, particularly of PP2A, removing the "signaling kinase brake", PP2A also fails to dephosphorylate PK and PDH, forming the "bottlenecks". (Black arrows = interrupted pathways).

The Figure 1 shows how tumors bypass the PK and PDH bottlenecks and evidently, the increase of glucose influx above the bottleneck, favors the supply of substrates to the pentose shunt, as pentose is needed for synthesizing ribonucleotides, RNA and DNA. The Figure 1 represents the stop below the citrate condensation. Hence, citrate quits the mitochondria to give via ATP citrate lyase, acetyl CoA and OAA in the cytosol of tumor cells. Acetyl CoA supports the synthesis of fatty acids and the formation of triglycerides. The other product of the ATP citrate lyase reaction, OAA, drives the transaminase cascade (ALAT and GOT transaminases) in a direction that consumes GLU and glutamine and converts in fine alanine into pyruvate and lactate plus NAD+. This consumes protein body stores that provide amino acids and much alanine (like in starvation). The Figure 1 indicates that malate dehydrogenase is a source of NAD+ converting OAA into malate, which backs-up LDH. Part of the malate converts to pyruvate (malic enzyme) and processed by LDH. Moreover, malate enters in mitochondria via the shuttle and gives back OAA to feed the citrate condensation. Glutamine will also provide amino groups for the "de novo" synthesis of purine and pyrimidine bases particularly needed by tumor cells. The Figure 1 indicates that ASP shuttled out of the mitochondrial, joins the ASP formed by cytosolic transaminases, to feed the synthesis of pyrimidine bases via ASP transcarbamylase, a process also enhanced in tumor cells. In tumors, this silences the argininosuccinate synthetase step of the urea cycle [18-20]. This blockade also limits the supply of fumarate to the Krebs cycle. The latter, utilizes the α ketoglutarate provided by the transaminase reaction, since α ketoglutarate coming via aconitase slows down. Indeed, NO and peroxynitrite increase in tumors and probably block aconitase. The Figure 1 indicates the cleavage of arginine into urea and ornithine. In tumors, the ornithine production increases, following the polyamine pathway. Ornithine is decarboxylated into putrescine by ornithine decarboxylase, then it captures the backbone of S adenosyl methionine (SAM) to form polyamines spermine then spermidine, the enzyme controlling the process is SAM decarboxylase. The other reaction product, 5-methlthioribose is then decomposed into methylthioribose and adenine, providing purine bases to the tumor. We shall analyze below the role of SAM in the carcinogenic mechanism, its destruction aggravates the process.

In summary, it is like if the mechanism switching from gluconeogenesis to glycolysis was jammed in tumors, PK and PDH are at rest, like for gluconeogenesis, but citrate synthase is on. Thus, citric acid condensation pulls the glucose flux in the glycolytic direction, which needs NAD+; it will come from the pyruvate to lactate conversion by lactate dehydrogenase (LDH) no longer in competition with a quiescent Pcarb. Since the citrate condensation consumes acetyl CoA, ketone bodies do not form; while citrate will support the synthesis of triglycerides via ATP citrate lyase and fatty acid synthesis... The cytosolic OAA drives the transaminases in a direction consuming amino acid. The result of these metabolic changes is that tumors burn glucose while consuming muscle protein and lipid stores of the organism. In a normal physiological situation, one mobilizes stores for making glucose or ketone bodies, but not while burning glucose! Tumor cell metabolism gives them a selective advantage over normal cells. However, one may attack some vulnerable points.

## II Starters for cancer metabolic anomaly

### 1. Lessons from oncogenes

Following the discovery of Rous sarcoma virus transmitting cancer [21], we have to wait the work of Stehelin [22] to realize that this retrovirus only transmitted a gene captured from a previous host. When one finds that the transmitted gene encodes the Src tyrosine kinase, we are back again to the tyrosine kinase signals, similar to those activated by insulin or IGF, which control carbohydrate metabolism, anabolism and mitosis.

An up regulation of the gene product, now under viral control causes tumors. However, the captured viral oncogene (v-oncogene) derives from a normal host gene the proto-oncogene. The virus only perturbs the expression of a cellular gene the proto-oncogene. It may modify its expression, or its regulation, or transmit a mutated form of the proto-oncogene. Independently of any viral infection, a similar tumorigenic process takes place, if the proto-oncogene is translocated in another chromosome; and transcribed under the control of stronger promoters. In this case, the proto-oncogene becomes an oncogene of cellular origin (c-oncogene). The third mode for converting a prot-oncogene into an oncogene occurs if a retrovirus simply inserts its strong promoters in front of the proto-oncogene enhancing its expression.

It is impressive to find that retroviral oncogenes and cellular oncogenes disturb this major signaling pathway: the MAP kinases mitogenic pathways. At the ligand level we find tumors such Wilm's kidney cancer, resulting from an increased expression of insulin like growth factor; we have also the erbB or V-int-2 oncogenes expressing respectively NGF and FGF growth factor receptors. The receptors for these ligands activate tyrosine kinase signals, similarly to insulin receptors. The Rous sarcoma virus transmits the src tyrosine kinase, which activates these signals, leading to a chicken leukemia. Similarly, in murine leukemia, a virus captures and retransmits the tyrosine kinase abl. Moreover, abl is also stimulated if translocated and expressed with the bcr gene of chromosome 22, as a fusion protein (Philadelphia chromosome). Further, ahead Ras exchanging protein for GTP/GDP, and then the Raf serine-threonine kinases proto-oncogenes are known targets for oncogenes. Finally, at the level of transcription factors activated by MAP kinases, one finds cjun, cfos or cmyc. An avian leucosis virus stimulates cmyc, by inserting its strong viral promoter. The retroviral attacks boost the mitogenic MAP kinases similarly to inflammatory cytokins, or to insulin signals, that control glucose transport and gycolysis.

In addition to the MAP kinase mitogenic pathway, tyrosine kinase receptors activate PI3 kinase pathways; PTEN phosphatase counteracts this effect, thus acting as a tumor suppressor. Recall that a DNA virus, the Epstein-Barr virus of infectious mononucleose, gives also the Burkitt lymphoma; the effect of the virus is to enhance PI3 kinase. Down stream, we find mTOR (the target of rapamycine, an immune-suppressor) mTOR, inhibits PP2A phosphatase, which is also a target for the simian SV40 and Polyoma viruses. Schematically, one may consider that the different steps of MAP kinase pathways are targets for retroviruses, while the different steps of PI3 kinase pathway are targets for DNA viruses. The viral-driven enhanced function of these pathways mimics the effects of their prolonged activation by their usual triggers, such as insulin or IGF; one then expects to find an associated increase of glycolysis. The insulin or IGF actions boost the cellular influx of glucose and glycolysis. However, if the signaling pathway gets out of control, the tyrosine kinase phosphorylations may lead to a parallel PK blockade [3-5] explaining the tumor bottleneck at the end of glycolysis. Since an activation of enyme kinases may indeed block essential enzymes (PK, PDH and others); in principle, the inactivation of phosphatases may also keep these enzymes in a phosphorylated form and lead to a similar bottleneck and we do know that oncogenes bind and affect PP2A phosphatase. In sum, a perturbed MAP kinase pathway, elicits metabolic features that would give to tumor cells their metabolic advantage.

### 2. The methylation hypothesis and the role of PP2A phosphatase

In a remarkable comment, Newberne [23] highlights interesting observations on the carcinogenicity of diethanolamine [24] showing that diethanolamine decreased choline derivatives and methyl donors in the liver, like does a choline deficient diet. Such conditions trigger tumors in mice, particularly in the B6C3F1 strain. Again, the historical perspective recalled by Newberne's comment brings us back to insulin. Indeed, after the discovery of insulin in 1922, Banting and Best were able to keep alive for several months depancreatized dogs, treated with pure insulin. However, these dogs developed a fatty liver and died. Unlike pure insulin, the total pancreatic extract contained a substance that prevented fatty liver: a lipotropic substance identified later as being choline [25]. Like other lipotropes, (methionine, folate, B12) choline supports transmethylation reactions, of a variety of substrates, that would change their cellular fate, or action, after methylation. In the particular case concerned here, the removal of triglycerides from the liver, as very low-density lipoprotein particles (VLDL), requires the synthesis of lecithin, which might decrease if choline and S-adenosyl methionine (SAM) are missing. Hence, a choline deficient diet decreases the removal of triglycerides from the liver; a fatty liver and tumors may then form. In sum, we have seen that pathways exemplified by the insulin-tyrosine kinase signaling pathway, which control anabolic processes, mitosis, growth and cell death, are at each step targets for oncogenes; we now find that insulin may also provoke fatty liver and cancer, when choline is not associated to insulin. We must now find how the lipotropic methyl donor controls the signaling pathway. We know that after the tyrosine kinase reaction, serine-threonine kinases take over along the signaling route. It is thus highly probable that serine-threonine phosphatases will counteract the kinases and limit the intensity of the insulin or insulin like signals. One of the phosphatases involved is PP2A, itself the target of DNA viral oncogenes (Polyoma or SV40 antigens react with PP2A subunits and cause tumors). We found a possible link between the PP2A phosphatase brake and choline in works on Alzheimer's disease [26]. Indeed, the catalytic C subunit of PP2A is associated to a structural subunit A. When C receives a methyle, the dimer recruits a regulatory subunit B. The trimer then targets specific proteins that are dephosphorylated [27]. In Alzheimer's disease, the poor methylation of PP2A is associated to an increase of homocysteine in the blood [26]. The result of the PP2A methylation failure is a hyperphosphorylation of Tau protein and the formation of tangles in the brain. Tau protein is involved in tubulin polymerization, controlling axonal flow but also the mitotic spindle. It is thus possible that choline, via SAM, methylates PP2A, which is targeted toward the serine-threonine kinases that are counteracted along the insulin-signaling pathway. The choline dependent methylation of PP2A is the brake, the "antidote", which limits "the poison" resulting from an excess of insulin signaling. Moreover, it seems that choline deficiency is involved in the L to M2 transition of PK isoenzymes [28].

### 3. Cellular distribution of PP2A

In fact, the negative regulation of Ras/MAP kinase signals mediated by PP2A phosphatase seems to be complex. The serine-threonine phosphatase does more than simply counteracting kinases; it binds to the intermediate Shc protein on the signaling cascade, which is inhibited [29]. The targeting of PP2A towards proteins of the signaling pathway depends of the assembly of the different holoenzymes. The carboxyl methylation of C-terminal leucine 309 of the catalytic C unit, permits to a dimeric form made of C and a structural unit A, to recruit one of the many regulatory units B, giving a great diversity of possible enzymes and effects. The different methylated ABC trimers would then find specific targets. It is consequently essential to have more information on methyl transferases and methyl esterases that control the assembly or disassembly of PP2A trimeric forms.

A specific carboxyl methyltransferase for PP2A [30] was purified and shown to be essential for normal progression through mitosis [31]. In addition, a specific methylesterase that demethylates PP2A has been purified [32]. Is seems that the methyl esterase cancels the action of PP2A, on signaling kinases that increase in glioma [33]. Evidently, the cellular localization of the methyl transferase (LCMT-1) and the phosphatase methyl esterase (PME-1) are crucial for controlling PP2A methylation and targeting. Apparently, LCMT-1 mainly localizes to the cytoplasm and not in the nucleus, where PME-1 is present, and the latter harbors a nuclear localization signal [34]. From these observations, one may suggest that PP2A gets its methyles in the cytoplasm and regulates the tyrosine kinase-signaling pathway, attenuating its effects. A methylation deficit should then decrease the methylation of PP2A and boost the mitotic insulin signals as discussed above for choline deficiency, steatosis and hepatoma. At the nucleus, where PME-1 is present, it will remove the methyl, from PP2A, favoring the formation of dimeric AC species that have different targets, presumably proteins involved in the cell cycle. It is interesting to quote here the structural mechanism associated to the demethylation of PP2A. The crystal structures of PME-1 alone or in complex with PP2A dimeric core was reported [35] PME-1 binds directly to the active site of PP2A and this rearranges the catalytic triad of PME-1 into an active conformation that should demethylate PP2A, but this also seems to evict a manganese required for the phosphatase activity. Hence, demethylation and inactivation would take place in parallel, blocking mitotic actions. However, another player is here involved, the so-called PTPA protein, which is a PP2A phosphatase activator. Apparently, this activator is a new type of cis/trans of prolyl isomerase, acting on Pro190 of the catalytic C unit isomerized in presence of Mg-ATP [36], which would then cancel the inactivation mediated by PME-1. Following the PTPA action, the demethylated phosphatase would become active again in the nucleus, and stimulate cell cycle proteins [37,38] inducing mitosis. Unfortunately, the ligand of this new prolyl isomerase is still unknown. Moreover, we have to consider that other enzymes such as cytochrome P450 have also demethylation properties.

In spite of deficient methylations and choline dehydrogenase pathway, tumor cells display an enhanced choline kinase activity, associated to a parallel synthesis of lecithin and triglycerides.

The hypothesis to consider is that triglycerides change the fate of methylated PP2A, by targeting it to the nucleus, there a methylesterase demethylates it; the phosphatase attacks new targets such as cell cycle proteins, inducing mitosis. Moreover, the phosphatase action on nuclear membrane proteins may render the nuclear membrane permeable to SAM the general methyl donor; promoters get methylated inducing epigenetic changes.

The relative decrease of methylated PP2A in the cytosol, not only cancels the brake over the signaling kinases, but also favors the inactivation of PK and PDH, which remain phosphorylated, contributing to the metabolic anomaly of tumor cells.

In order to prevent tumors, one should then favor the methylation route rather than the phosphorylation route for choline metabolism. This would decrease triglycerides, promote the methylation of PP2A and keep it in the cytosol, reestablishing the brake over signaling kinases. Moreover, PK, and PDH would become active after the phosphatase action. One would also gain to inhibit their kinases as recently done with dichloroacetate for PDH kinase [17]. The nuclear or cytosolic targeting of PP2A isoforms is a hypothesis also inspired by several works [34,36-38].

### 4. Hypoxia is an essential issue to discuss

We know that the transition from fetal to adult hemoglobin, with a lower oxygen affinity, would repeat the phylogenic adaptation of "aquatic creatures" to breath in air and live on land. Many adequate "adult proteins" replace their fetal isoform: muscle proteins utrophine, switches to dystrophine; enzymes such as embryonic M2 PK [39] is replaced by M1. Hypoxic conditions seem to trigger back the expression of the fetal gene packet via HIF1-Von-Hippel signals. The mechanism would depend of a double switch since not all fetal genes become active after hypoxia. First, the histones have to be in an acetylated form, opening the way to transcription factors, this depends either of histone deacetylase (HDAC) inhibition or of histone acetyltransferase (HAT) activation, and represents the main switch. Second, a more specific switch must be open, indicating the adult/fetal gene couple concerned, or more generally the isoform of a given gene that is more adapted to the specific situation. When the adult gene mutates, an unbound ligand may indeed indicate, directly or indirectly, the particular fetal copy gene to reactivate [40]. In anoxia, lactate is more difficult to release against its external gradient, leading to a cytosolic increase of up-stream glycolytic products, 3P glycerate or others. These products may then be a second signal controlling the specific switch for triggering the expression of fetal genes, such as fetal hemoglobin or the embryonic M2 PK; this takes place if histones (main switch) are in an acetylated form. This is the case if HAT is active in tumor cells, in spite of the decrease of butyrate a HDAC inhibitor. In tumor cells, one finds hypermethylated regions, in which HDAC activity silences a set of genes and hypomethylated parts in which HAT activates other genes.

## III. Growth hormone-IGF actions, the control of asymmetrical mitosis

When IGF - Growth hormone operate, the fatty acid source of acetyl CoA takes over. Indeed, GH stimulates a triglyceride lipase in adipocytes, increasing the release of fatty acids and their β oxidation. In parallel, GH would close the glycolytic source of acetyl CoA, perhaps inhibiting the hexokinase interaction with the mitochondrial ANT site. This effect, which renders apoptosis possible, does not occur in tumor cells. GH mobilizes the fatty acid source of acetyl CoA from adipocytes, which should help the formation of ketone bodies, but since citrate synthase activity is elevated in tumors, ketone bodies do not form. Hence, butyrate cannot inhibit histone deacetylase (HDAC), the enzyme cuts acetylated histone tails, this will silence several genes like PETEN, P53, or methylase inhibitory genes. It is probable that the IGFBP gene gets silent as well. IGFBP is a protein that binds IGF with high affinity and controls its effects [41]. In parallel, GH hormone induces in the liver, the synthesis and release of insulin like growth factor (IGF). The latter, activates like insulin, the IGF-tyrosine kinase receptors (IGFR), triggering the MAP kinase-ERK mitogenic signal. The surface distribution of IGF-IGFR may determine if a cell is sterile or endowed with a mitotic potential. We propose that this distribution depends of IGFBP; suppose that a cell starting mitosis secretes IGFBP at one pole, and that IGFBP binds to the extracellular matrix, forming patches of IGFBP. The patch may attract the IGF-IGFR complex by a capping process. The daughter cell that inherits the IGFR receptor patch becomes a stem cell, while the cell poor in IGFR will be sterile and follow the differentiation program.

This does not mean that the sterile daughter cell, at rest in the Go phase, cannot exit and divide again; this would require a reactivation of IGFR receptor genes in order to build up again the membrane concentration of IGFR.

## IV. Compounds for correcting tumor metabolism

The figure 1 indicates interrupted and enhanced metabolic pathways in tumor cells.

In table 1, the numbered pathways represent possible therapeutic targets; they cover several enzymes. When the activity of the pathway is increased, one may give inhibitors; when the activity of the pathway decreases, we propose possible activators.

**Table 1:** Therapeutic targets

Targets	Metabolic pathway	Activity	Drugs: inhibitors or activators	References
**1**	Proteolysis/alanine/transaminase/pyruvate	**increased**	aminooxacetic, 2PAM, D alanine, β chloro-L-alanine	[42,43]

**2**	Lactate dehydrogenase/pyruvate/lactate/NAD+ Malate dehydrogenase/OAA/malate/NAD+ Malic enzyme/malate/pyruvate/lactate/NAD+	**increased** **increased** **increased**	Br pyruvate, gossypol D malate, humic acid D malate	[44-46]

**3**	Glycolysis	**increased**	mannoheptulose, lodinamineepalresta, citrate	[47-49]

**4**	PEP carboxykinase/PEP/OAA Pyruvate carboxylase/pyruvate/OAA	**increased** **decreased**	Cl-PEP, β sulphopyruvate 3-hydroxybutyrate (ketone bodies will activate)	[50,51] [52]

**5**	Pyruvate kinase (M2 bottleneck)/PEP/pyruvate Pyruvate dehydrogenase/pyruvate/acetylCoA	**decreased** **decreased**	dihydroxyphenylethanol, polyethylene - glycol lipoic acid, kinase inhibitors (dichloroacetate, 2-chloropropionate) or PP2A agonists (xylulose5P, B12, choline)	[53-55] [16,17]

**6**	Lipolytic fatty acid source of acetyl CoA Carnitine transporter	**increased**	niacine, Growth hormone inhibitors Avoid oxfenicine or levofloxacin	[56,57]

**7**	Citrate synthase (OAA+ acetylCoA)/citrate	**increased**	Dserine, fluoroacetylCoA, carboxymethyl-CoA, citrate (via product inhibition), capsaicine (via electron transport inhibition and NADH increase)	[13]

**8**	ATPcitrate lyase citrate/OAA+acetylCoA Triglycerides synthesis via acetyl CoA Other OAA sources	**increased** **increased** **increased**	hydroxycitrate, fluorocitrate xanthohumol (removes triglycerides) D malate	[58] [59]

**9**	Choline dehydrogenase/methylation/lipotropic Choline kinase/phosphorylcholine/lecithin	**decreased** **increased**	choline miltefosin, farnesol	[60,61]

**10**	PP2A methylation (it counteracts signaling and enzyme kinases)	**decreased**	betaine, folate, B12, trimethylglycine (via SAM)	

**11**	Histone deacetylase (gene silencing)	**increased**	butyrate, valproate, benzamide, trichostatin	[62]

**12**	Cytochrome P450 demethylase (hypomethylated promoters)HAT/hexokinase expression Histone acetylase	**increased** **increased**	ketonazole, bergamottin, quinine anacardic acid, garcinol, curcumine	[63-67] [66,67]

**13**	Tyrosine kinase-signaling route (MAP kin, PI3 kin, PLCγ)	**increased**	imatinib mésilate (glivec chemotherapy)	

**14,15, 16**	GH-IGF IGFBP	**increased** **decreased**	octreotide, pegvisomant apigenin, casodex, omeprazol	[68-70]

**17**	Arginine dependency	**increased**	arginine deprivation diet, pegylated arginine deiminase	[18-20] [71-73]

**18**	Arginase/arginine/ornithine	**increased**	norvaline, N-omega-hydroxy-nor-arginine, boroargininre	[74-76]

**19**	Polyamine pathway: Ornithine decarboxylase/putrescine SAM decarboxylase	**increased**	DFMO (2-difluoromethylornithine), MGBG methylglyoxal bis (guanylhydrazone), 4-amidinoindan-1one-2'-amidinhydrazone(alfa-fluoromethylhistidine)	[77-79]

**20**	Glutaminase/glutamate	**increased**	DON(6-diazo-5-oxo-l-norleucine), riluzole, naftazone	[80].

**21**	Argininosuccinate synthetase	**decreased**	troglitazone	[81]

## V. Cancer prevention, immune destruction of tumor cells

### 1. A preventive cure

André Gernez [82-84] applied for the first time the stem cell concept to cancer and neurological diseases. "He compared tissues to colonies of bees in which, only the queens renew the colony, while most bees are sterile workers". For preventing cancer, he imagined an annual cure, which did not receive much attention. It consists of three points: **first **a fasting period once a year, "this is recommended by most religions", **second **one should eat more fruits, vegetables, less meat, consume products rich in magnesium, vitamins C, E, selenium, this is now currently accepted. The **third **point is more difficult to apply, without testing it on animal models. It aims to kill once a year eventual tumor cells, by giving for a few days an anti-mitotic, colchicine for example. Gernez mentions that chloral, anciently used for treating mental patients, also protected them from cancer, because it has anti-mitotic properties. The idea being to kill the very first tumor cells that eventually appear, and if missed the first year, one would catch them the next year etc... How can we explain the possible prevention? The first point is evident, fasting increases ketone bodies such as butyrate, which is a histone deacetylase (HDAC) inhibitor, this keeps histone tails acetylated, which cancels the silencing of several genes starting the tumor process. The second part of the procedure takes advantage of the anti-inflammatory properties of flavonoids found in fruits and vegetables. Flavonoids cancel the effects of hypoxia on the expression of a set of genes: cyclooxygenase (COX), VEGF, NOsynthase, glycolytic enzymes, induction of fetal genes. Recall that fetal M2 PK expression is a typical feature of tumor cells. We know the anti-tumor effect of avastin and anti VEGF antibodies. The anti-cancer properties of 'non-steroidal anti-inflammatory drugs,'' NSAIDS, results from the inhibition of COX decreasing pro-inflammatory prostanoids. Moreover, magnesium may inhibit calcium dependent phospholipases forming arachidonic acid, the prostanoid precursor and diacylglycerol (DAG) with mitogenic effects. In addition, vitamins C, E, and selenium, quench superoxides and peroxynitrite, formed by a poor oxygen reduction during hypoxia. One may strengthen this second line of protection with methyl donors, folate, and vitamin B_12. _They would attenuate, via methylated PP2A, the signaling kinases, activated by insulin or IGF, and regulate the supply of glucose, anabolism and mitosis. The third point deserves additional experiments. The hypothesis is that tumor cells appear frequently, but the immune surveillance constantly eliminates them. If the immune surveillance fails, an anti-mitotic would kill once a year, tumor cells before they reach a critical mass. This third point of the procedure would back-up the immune surveillance when it fails, but deserves an evaluation.

### 2. The immune surveillance

Tumor cells or virus-invaded cells would display lower MHC-1 levels, the major histocompatibility complex, which is a self-recognition device. The consequence of the MHC-1 decrease is to trigger the "Natural killer" (NK) immune protection, because MHC-1 stops neutralizing receptors (KIR) on NK cells, which triggers the release of perforines by NK cells. The perforines kill cells poor in MHC-1. Well, an up-regulation of Insulin or IGF signals decreases MHC-1, and may then trigger the NK protection [85], if such a protection fails then tumor cells may survive...

The NK protection is activated by leucotriene LTB4 formed by lipoxygenase, and inhibited by the fever prostaglandin PGE2 formed by cycloxygenase [86]. Hence, inhibitors of PGE2 and cycloxygenase trigger the NK protection; this is an effect of non-steroid anti-inflammatory drugs (NSAIDS) aspirin, ibuprofen, or of some histone acetylase (HAT) inhibitors (curcumin, anacardic acid garcinol etc...). It is also particularly interesting to increase serotonin (5HT) that neutralizes the inhibition of NK cells by PGE2. Echinacea extracts [87]; 5HT uptake inhibitors (St John's Wort tea, prozac, melatonine) [88] boost the release of 5HT from platelets, particularly if platelets are full of 5HT before reaching the site. A substance P antagonist (aprepitan) [89] avoids their premature degranulation). Finally 5HT can be preserved from its enzymatic conversion into quinolinic acid by inhibiting indoleamine,2,3-dioxygenase with exiguamine or 1-methytryptophane [90,91]. Echinacea, melatonin, 5HT uptake inhibitors, substance P antagonists, and HAT inhibitors [66,67,92,93] boost NK mediated protection against eventual tumor cell, or viral infections.

## VI. A Possible sequential plural-therapy

As a complement to radiotherapies or chemotherapies, we propose to try, after validating it on animal models a sequential metabolic therapy. 1- Inhibit alanine transaminase, glutaminase, ornithine decarboxylase, arginase; and decrease alanine, glutamine, arginine, supplies. 2- Open PK and PDH bottlenecks (enzyme kinase inhibition and PP2A phosphatase activation by methylation helpers) 3- Close citrate synthase, ATP citrate lyase, increase the NADH mitochondrial potential. 4- Decrease GH/IGF [94] and "tyrosine kinase receptor" signals. 5- Cancel epigenetic changes using HDAC inhibitors and then HAT inhibition. 6- Boost immune NK protection.

In this review, we have collected observations on tumor cell metabolism obtained by many laboratories working on cancer for the past 80 years. When we find that choline related methylations protect from liver cancer, that PP2A methylations controls the Tyrosine kinase oncogenic signals, activated by IGF-GH it seemed logic to establish a link between these observations even if they were not obtained simultaneously. The impression is that the link we make is perhaps artificial. However, a reference for all the experimental observations is given. The link between them is the interpretation we propose. This model has inspired our recent works, and convergent interpretations from other laboratories support some of the model predictions. See for example reference [95] suggesting a therapeutic approach with multiple drugs acting on glycolysis. Additional works target with drugs this metabolic transformation of tumor cells [96] and manipulate for example α ketoglutarate dehydrogenase [97] an enzyme similar to PDH, which is less active in tumor cells as indicated in the model. Other laboratories attack the increased glycolysis with bromopyruvate as we also suggest [98]. As for the spectacular effects of citrate and isocitrate [99,100] or octreotide and capsaicine that the model predicts, they may find a therapeutic application beneficial to patients as shown by experiments on animals. Finally, the recent discovery of a population of Dwarfs with no GH receptors, which do not develop cancers [101], illustrates the GH/IGF prediction, establishing a link between ancient and recent biochemical observations on tumors. By adding octreotide a somatostatin analogue, to the lipoic acid isocitrate mixture, our recent results (in press) seem to validate the presented model.

## Conclusion: The origin of Cancers by means of metabolic selection

The disruption of cells by internal or external compounds, releases substrates stimulating the tyrosine kinase signals for anabolism proliferation and stem cell repair, like for most oncogenes. If such signals are not limited, there is a parallel blockade of key metabolic enzymes by activated kinases or inhibited phosphatases. The result is a metabolism typical of tumor cells, which gives them a selective advantage; stabilized by epigenetic changes. A proliferation process, in which the two daughter cells divide, increases the tumor mass at the detriment of the body. Inevitable mutations follow, selecting the most robust tumor cells... We only hope that a non-toxic mixture will bring back to normality cells when they acquire the selective metabolic advantage, which leads them to cancer, this mixture may back-up current cancer therapies.

## Competing interests

The authors declare that they have no competing interests.

## Authors' contributions

MI elaborated the biochemical model for cancer metabolism and drafted the manuscript. LS tested model predictions for drugs affecting selectively tumors. All authors read and approved the final manuscript
